# Longitudinal analysis of healthy colon establishes aspirin as a suppressor of cancer-related epigenetic aging

**DOI:** 10.1186/s13148-020-00956-9

**Published:** 2020-11-03

**Authors:** Faiza Noreen, Anna Chaber-Ciopinska, Jaroslaw Regula, Primo Schär, Kaspar Truninger

**Affiliations:** 1 Department of Biomedicine, University of Basel, Mattenstrasse 28, 4058 Basel, Switzerland; 2 Swiss Institute of Bioinformatics, 4053 Basel, Switzerland; 3 Department of Gastroenterology, Medical Center for Postgraduate Education, Maria Sklodowska-Curie Memorial Cancer Center, Warsaw, Poland; 4 Gastroenterologie Oberaargau, 4900 Langenthal, Switzerland

**Keywords:** Colon cancer, Aspirin, Aging, DNA methylation, Epigenetics

## Abstract

**Background:**

Colon cancer (CC) is the third most common cancer worldwide, highlighting the importance of developing effective prevention strategies. Accumulating evidence supports that aspirin use reduces CC incidence. We reported previously that aspirin suppresses age-associated and CC-relevant DNA methylation (DNAm) in healthy colon. Here we addressed the aspirin’s effectiveness in longitudinal cohort.

**Methods:**

We measured genome-wide DNAm in 124 healthy normal mucosa samples taken at baseline (time point 1, t1) and after 10-years follow-up (time point 2, t2) from a longitudinal female screening cohort. We investigated the time-dependent methylation drift in aspirin users and nonusers using multivariable regression and related the modulatory effect of aspirin to colonic epigenome-aging and CC.

**Results:**

Over time, compared to nonusers, long-term (≥ 2 years) aspirin users showed less hypermethylated CpGs (proximal: 17% vs. 87%; distal: 16% vs. 70%) and more hypomethylated CpGs (proximal: 83% vs. 13%; distal: 84% vs. 30%). Overall, users showed 2% (*P* = 0.02) less mean methylation levels than nonusers in proximal colon and displayed repressed methylation age (mAge). Methylation loss in users occurred at several CC-specific tumor suppressors that gained methylation in nonusers. Methylation loss in users effected genes involved in immune system and inflammation, while methylation gain in nonusers effected genes involved in metabolism.

**Conclusions:**

This is the first longitudinal study demonstrating effectiveness of aspirin-use in suppression of age-related and CC-relevant hypermethylation in the normal colon. These findings provide a rationale for future studies to evaluate loci that may serve as markers to identify individuals that will benefit most from aspirin and hence increase its efficiency in CC prevention and therapy.

## Background

Colon cancer (CC) is the third most common cancer worldwide and the second most common in terms of cancer-related mortality [1]. Age is the strongest risk factor for sporadic CC [2]; however, the risk varies among individuals, even within the same age group, which may reflect heterogeneity in exposure to environmental factor. Indeed, several observational studies have shown that diet and lifestyle are associated with increase CC risk [2–4]. Thus, primary prevention by modification of environmental and lifestyle exposures and the use of chemoprevention are important strategies to reduce further CC incidence. Among chemopreventive agents, long-term use of aspirin is associated with decreased risk of CC incidence, mortality and cancer recurrence after therapy [5–7]. Understanding aspirin’s mechanism of action is important to increase its benefit-risk ratio, given the known hazards of its use [8–10]. While the inhibition of cyclooxygenases (COX) is well characterized as an underlying cause [11], several COX-independent mechanisms of aspirin, such as suppression of cellular proliferation and induction of apoptosis, inhibition of WNT/β-catenine and NF-kB signaling have been suggested to play a role [12–14]; however, the impact of aspirin use on epigenome as potential mechanism is poorly studied.

Epigenetic modifications, particularly DNA methylation (DNAm) changes, are recognized as one of the most common molecular alterations in human tumors, including CC [15]. CC-subtype-specific DNAm are readily detectable in healthy normal mucosa [16–18], before the occurrence of visible precursor lesions, which ultimately may progress to CC [19]. The stability of DNAm on as molecular marker and its effect on gene expression [20] facilitates its clinical use in early cancer detection and makes it a potential target to predict treatment outcome and patient’s response to therapy.

We have previously shown in a cross-sectional study that aspirin modulates age-associated DNAm changes in the colon and, thereby, CC risk [17]. Understanding how aspirin controls the stability of DNAm in aging tissue is of both biological and clinical importance. Hence, the present study investigated the effect of aspirin use on DNAm in a longitudinal screening female cohort and its effect on human biological aging in normal colon using three established epigenetic clocks; Horvath’s [21], Hannum’s [22] and PhenoAge [23]. Finally, we assessed the functional link between suppressive gene methylation by aspirin use and their link to CC risk.

## Results

**Fig. 1:**
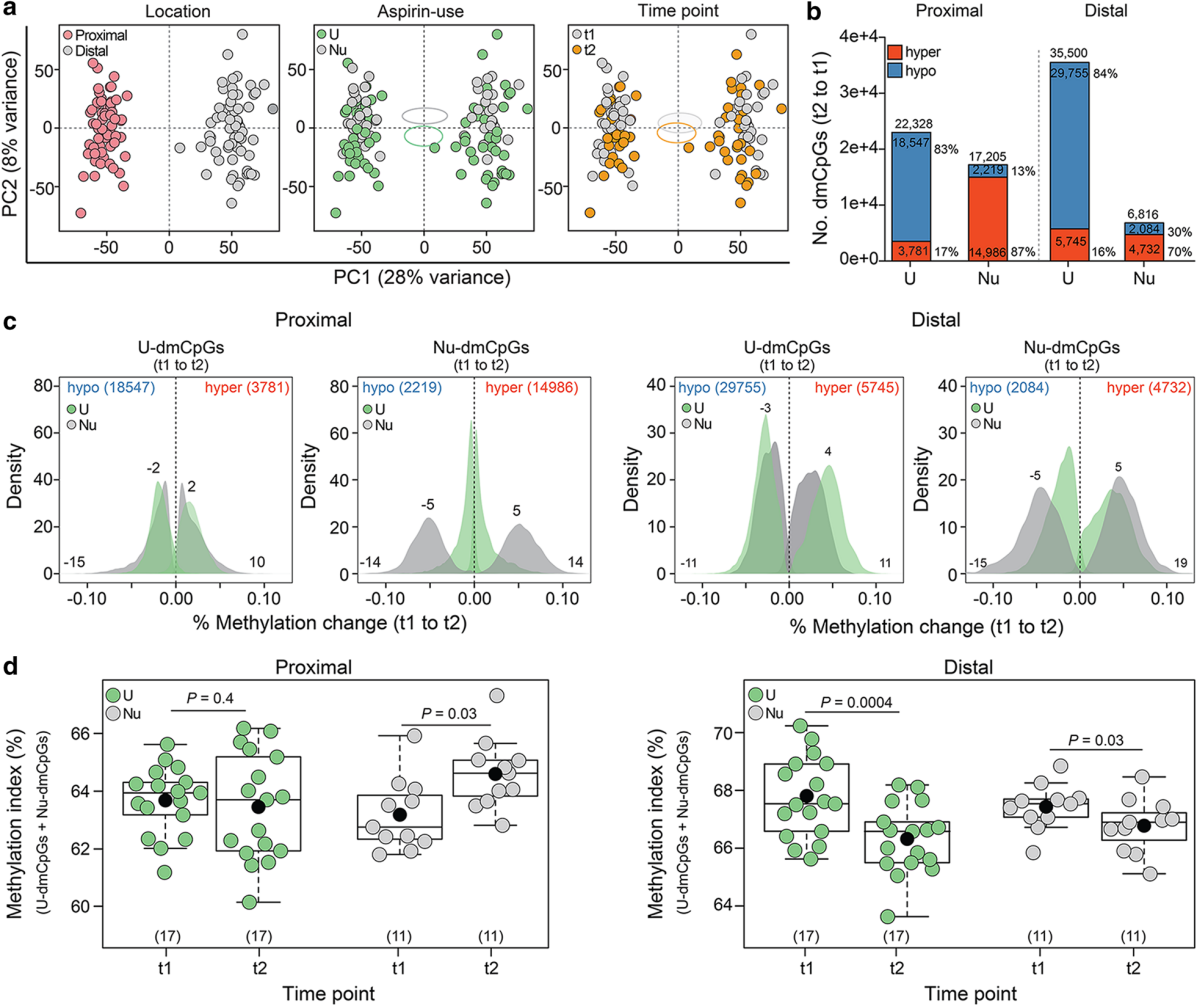
Genome-Wide DNA methylation in aspirin users and nonusers. **a** PCA for 10,000 most variable CpGs across 124 colon biopsies by colon location or by aspirin use (U: users, Nu: nonusers) and by time points (baseline: t1, 10 years follow-up: t2). Shown are the principal component 1 (PC1) on x- and PC2 on y-axes. **b** Number of unique U- and Nu-dmCpGs in proximal and distal colon; hypermethylated (hyper), hypomethylated (hypo). Benjamini-Hochberg (BH) false discovery rate adjusted *P* values (adj. *P*) < 0.05 were used as cutoff to identify significant dmCpGs. **c** Percentage (%) methylation change (t2 vs. t1) at U- and Nu-dmCpGs in users and nonusers for both colon locations. Maximum and median values are shown on the graph. **d** Methylation index (MI) for each subject, as the percentage mean methylation across all dmCpGs (Nu-dmCpGs + U-dmCpGs). Each circles represent one subject. For paired analysis, three individuals who were aspirin nonusers at t1 but became users in t2 were excluded from this analysis. Number of individual in each group is mentioned at the bottom. Shown are medians (line) and mean (black circle). *P* values by paired Wilcoxon signed rank test

To assess global changes in the DNAm over time, we subjected 124 healthy normal mucosa samples of screening females to Illumina infinium MethylationEPIC BeadChip. The major clinical characteristics of the participants are summarized in Tables 1 and 2. Principal component analysis (PCA) on methylation profiles separated proximal from distal colon samples (28% variation), aspirin users (U) from nonusers (Nu) and/or t1 from t2 (8% variation) (Fig. 1a). Multivariable linear regression model was used to measure significant differentially methylated CpGs in aspirin users (U-dmCpGs) and in nonusers (Nu-dmCpGs) from t1 to t2. We identified more hypomethylated U-dmCpGs in aspirin users and more hypermethylated Nu-dmCpGs in nonusers (Fig. 1b, Additional file 1: Figure S1a). At U-dmCpGs, median methylation change (hyper and hypo) was generally similar in users and nonusers, while at Nu-dmCpGs, it was lower in users than nonusers (Fig. 1c). A methylation index (MI), designating the percentage of mean methylation across all dmCpGs, was calculated for each individual. In proximal colon, over time, the MI was increased in nonusers (*P* = 0.01), but not in users, which at t2 showed 2% (*P* = 0.02) decreased MI in users vs nonusers. In distal colon, the MI was decreased independently of aspirin use (Fig. 1d).

**Table 1:** Clinical characteristics of the study population

Characteristic	No. subjects	
	Time point 1 (t1)^a^	Time point 2 (t2)^b^
Aspirin regular use^c^		
Nonuser^d^	14 (45%)	11 (35%)
Long-term user (≥ 2 year)^e^	17 (55%)	20 (65%)
Age	50–70 (median 55)	60–80 (median 65)
BMI^f^		
Normal (18.5–25)	16 (52%)	14 (45%)
High (≥ 26)	15 (48%)	17 (55%)
Polyps		
No polyps	18 (58%)	23 (74%)
Yes	13 (42%)	8 (26%)
Proximal^g^		
Tubular adenoma	3 (23%)	1 (12.5%)
Serrated lesion^h^	1 (8%)	0 (0%)
Hyperplastic	1 (8%)	0 (0%)
Serrated adenoma	0 (0%)	0 (0%)
Mixed	0 (0%)	1 (12.5%) TA and SA
distal^i^		
Tubular adenoma	4 (31%)	4 (50%)
Serrated lesion	5 (38%)	0 (0%)
Hyperplastic	4 (31%)	0 (0%)
Serrated adenoma	1 (7%)	0 (0%)
Mixed	0 (0%)	1 (12.5%) TA and HP
Proximal and distal	0 (0%)	1 (12.5%) TA

^a^Baseline time point

^b^10-year follow-up

^c^Regular use defined as ≥ 2 tablets/week for ≥ 1 month

^d^Nonuser: women who indicated that they did not use the aspirin ≥ 2 tablets/week for ≥ 1 month (“minimum level”)

^e^Long-term user: women who indicated that they used the aspirin ≥ 2 tablets/week for ≥ 2 years

^f^BMI: body mass index; height (cm) and weight (kg) were self-reported and BMI was calculated (kg/m^2^) from these variables

^g^Proximal: cecum

^h^Serrated lesion: any serrated polyp including hyperplastic and serrated adenoma

^i^Distal: sigmoid colon

**Table 2:** Clinical characteristics of the study population based on aspirin regular use

Characteristics	No. subjects			
	Time point 1 (t1)^a^		Time point 2 (t2)^b^	
	Nonuser^c^	Long-term user (≥ 2 year)^d^	Nonuser^c^	Long-term user (≥ 2 year)^d^
	14 (45%)	17 (55%)	11 (35%)	20 (65%)
Age	50–70 (median 50)	51–69 (median 60)	60–77 (median 60)	60–80 (median 70)
BMI^e^				
Normal (18.5–25)	6 (43%)	10 (59%)	3 (27%)	11 (55%)
High (≥ 26)	8 (57%)	7 (41%)	8 (73%)	9 (45%)
Polyps				
No Polyps	5 (36%)	13 (76%)	10 (91%)	13 (65%)
Yes	9 (64%)	4 (24%)	1 (9%)	7 (35%)
Proximal^f^	3 (33%)	1 (25%)	0 (0%)	2 (29%)
Distal^g^	6 (67%)	3 (35%)	1 (100%)	4 (57%)
Proximal and distal	0 (0%)	0 (0%)	0 (0%)	1 (14%)

Regular use defined as ≥ 2 tablets/week for ≥ 1 month

^a^Baseline time point

^b^10-year follow-up

^c^Nonuser: women who indicated that they did not use the aspirin ≥ 2 tablets/week for ≥ 1 month (“minimum level”)

^d^Long-term user: women who indicated that they used the aspirin ≥ 2 tablets/week for ≥ 2 years

^e^BMI: body mass index; height (cm) and weight (kg) were self-reported and BMI was calculated (kg/m^2^) from these variables

^f^Proximal: cecum

^g^Distal: sigmoid colon

**Fig. 2:**
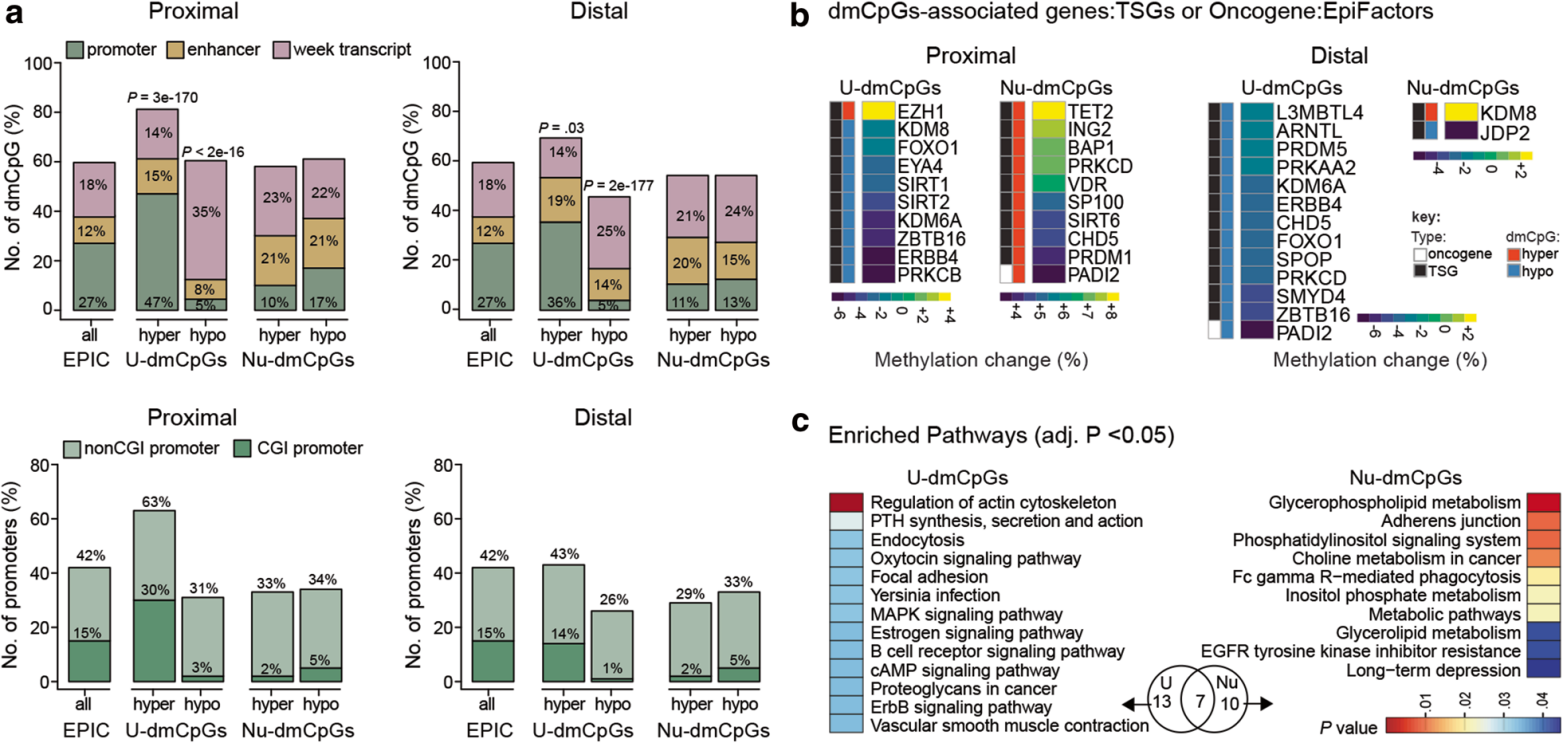
Functional analysis of dmCpGs. **a** Number of U- and Nu-dmCpGs at promoters, enhancers and weak-transcripts (top), promoters with and without CpG Island (CGI) (bottom). *P* values by fisher’s test. **b** Percentage (%) methylation change (t2 vs. t1) on U- and Nu-dmCpGs associated genes that are TSGs (black) or oncogenes (white) and are epigenetic regulators. **c** Pathways enriched in U- and Nu-dmCpGs (hyper and hypo) associated-genes found in proximal and distal colon. Venn diagram shows overlap of pathways (13 unique in users, 10 unique in nonusers, 7 common), and heatmaps illustrate the significance of enrichment (adj. *P* values < 0.05)

Enrichment analyses showed that in both colon locations dmCpGs were generally under-represented at CpG-island (CGI) promoters. Hypermethylated U-dmCpGs, however, were enriched at promoters regardless of the CGI status and hypomethylated U-dmCpGs were enriched at weakly transcribed regions according to their chromatin state defined by ChromHMM (Fig. 2a). The functional significance of genes associated with dmCpGs was investigated by overlapping them with tumor suppressor genes (TSG) and oncogenes known to be aberrantly expressed in CC [24, 25]. Notably, genes associated with hypomethylated U-dmCpGs and hypermethylated Nu-dmCpGs involved several TSG (Additional file 1: Figure S1b). Among them were genes that regulate DNA and histone methylation, e.g., (KDM8, SIRT1/2, CHD5). These findings suggest a widespread modulatory effect of aspirin on the colonic epigenome (Fig. 2b). Genes associated with U-dmCpGs (hyper and hypo) were allocated in 13 pathways involved in actin cytoskeleton, immune system (estrogen signaling) and inflammation (PI3K-Akt and cAMP signaling). Genes associated with Nu-dmCpGs (hyper and hypo) were enriched in 10 pathways primarily involved in metabolism (Fig. 2c*)*. Genes associating with both U- and Nu-dmCpGs were enriched in 7 pathways, including platelet activation and chemokines signaling pathways (Fig. 2c).

**Fig. 3:**
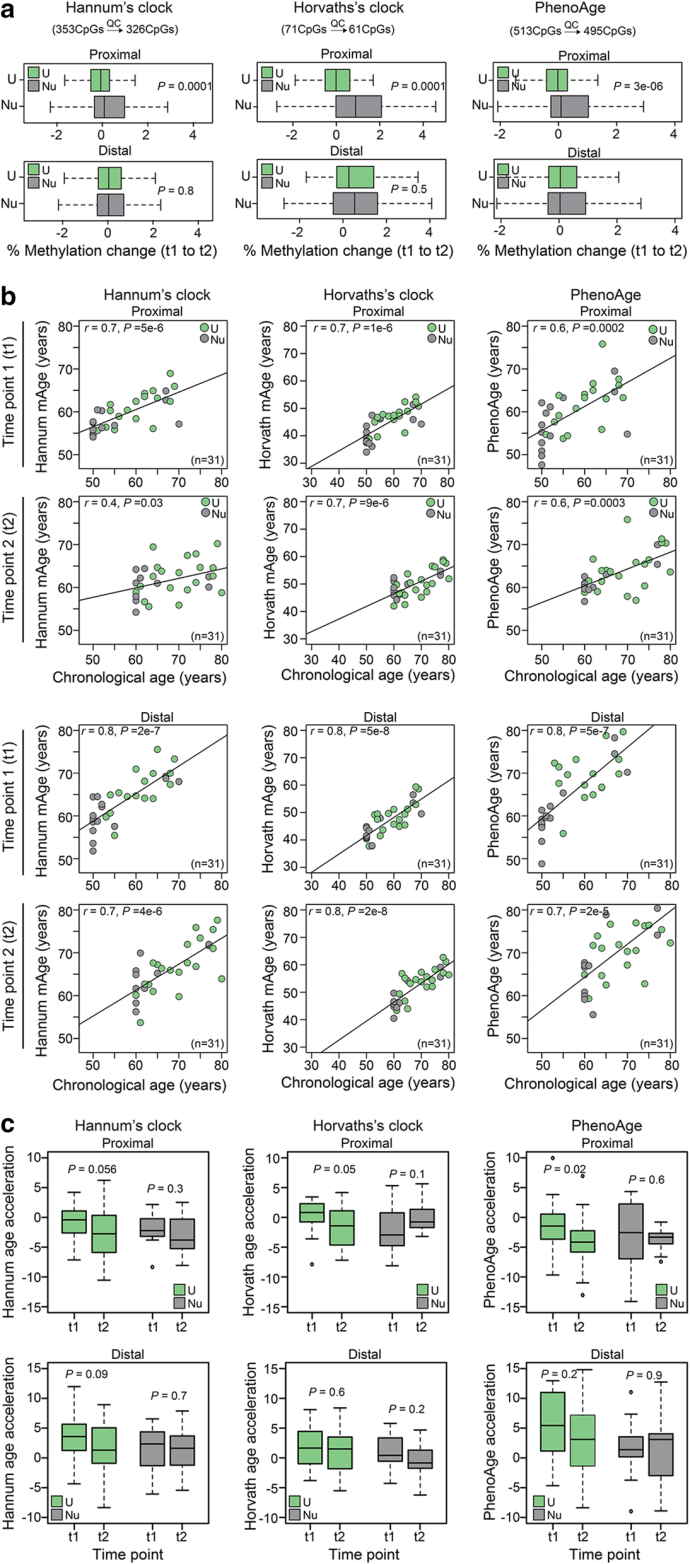
Epigenetic age deceleration in aspirin users. **a** Percentage methylation change on blood-based Hannum’s (61CpGs that passed QC out of 71CpGs), multi-tissue-based Hovath’s (326CpGs that passed QC out of 356CpGs) and blood-based phenoAge (495CpGs that passed QC out of 513CpGs) aging biomarkers. *P *values by Wilcoxon rank sum test. QC: quality control. **b** Correlation of the three epigenetic age estimates in the normal colon with the chronological age of the individual. The raw beta values without masking probes based on a detection *P* value were submitted. Separate plots are shown for each time point (t1 and t2) for proximal (top) and distal (bottom) samples. Shown are the person correlation coefficient and associated *P* values. **c** Distribution of epigenetic age acceleration in aspirin users and nonusers over time

Next, we asked whether the long-term aspirin use has an impact on methylation age (mAge). We utilized previously established three mAge estimators; Hannum (71 CpGs), Horvath (353 CpGs) and PhenoAge (513 CpGs). Overall, the median methylation change on CpGs corresponding to three mAge estimators was significantly reduced in users (Hannum: *P* = 0.0001; Horvath: *P* = 0.0001; PhenoAge: *P* = 3e−06) versus nonusers in proximal colon (Fig. 3a). No difference was found in distal colon. Further, there was moderate to high correlation between chronological age and mAge for all three estimators (*r* = 0.6–0.8); however, the Horvath based mAge was substantially lower than chronological age (Fig. 3b). To examine if the biological age varies between aspirin users and nonusers, we used age acceleration residuals (AAR). The residuals were obtained from regressing mAge on chronological age of all 124 samples; hence, they adjust for the chronological age bias between aspirin users and nonusers. Over time, in aspirin users, we found significant deceleration of Horvath mAge (*P* = 0.05) and PhenoAge (*P* = 0.02), and the deceleration trend was the same for Hannum age (*P* = 0.056) (Fig. 3c). Taken together, over time, aspirin users show low methylation levels and display decelerated mAge in proximal colon.

## Discussion

We conducted a 10-year follow-up study of our previous female screening cohort, finding the evidence that long-term aspirin use suppresses DNAm even after 10 years of its initial use and is associated with decelerated biological aging and CC risk.

The repressed DNAm was not limited to colon but was also found on aging-associated CpGs derived from multi-tissue and blood, indicating aspirin’s beneficial role beyond tissue type. We recognize that there are baseline DNAm differences between aspirin users and nonusers at t1 that may reflect different age and/or disease risks and reasons for aspirin use; however, baseline DNAm levels and age was corrected in our analysis. The consistency of our findings across Horvath [21], Hannum [22] and PhenoAge [23] defined age-associated CpGs implies that the observed suppression of DNAm in aspirin users is unlikely an artifact of the current data set. Our findings are in accordance with previous studies suggesting that epigenetic modifications are involved in the chemopreventive actions of aspirin. Recently, an association between aspirin use and *BRCA1* promoter methylation that affects the mortality after breast cancer has been shown [26]. Similarly, in mouse inflammation model, aspirin has been shown to reduced tumor multiplicity by suppressing histone deacetylases (HDAC), with concomitant increase of H3K27ac in the promoter regions of pro-inflammatory genes [27].

Previously in a cross-sectional screening female’s cohort, we found that aspirin represses rate of DNAm change in both colon locations. However, in the present longitudinal study, we found overall repressed MI in proximal but not in distal colon. The location-specific effect of aspirin was consistent with the repressed mAge in users in proximal colon for all three epigenetic clocks, but not in distal colon. These results are in line with previous reports showing daily aspirin intake reduces CC incidence and mortality and have a greater effect on CC of the proximal colon than on the distal colon [28]. Further evaluation for colon location dependence is needed with large sample size.

We found decelerated mAge using PhenoAge clock in aspirin users, although the same trend was found for Hannum’s and Horvath’s clock as well. While the accelerated mAge has been identified as a potential risk factor for age-related diseases and all-cause mortality [21, 23], the decelerated aging is expected to reduce disease incidence at any given age. The effect of aspirin on decelerated mAge in the present study is consistent with previous report showing increased lifespan of male mice upon aspirin use [29]. The mechanism(s) that drive deviation of mAge from chronological age are independent of cell proliferation, as these clocks operate in nonproliferation tissue (e.g., brain) as well as proliferating tissue (e.g., peripheral blood leukocytes) [21, 22].

There are several limitations to the current study. First is its small sample size. Second is the observational design of the study cannot imply that observed suppression in DNAm was caused by aspirin use. However, we adjusted for the potential confounders and still have large effects. Third is one important but unresolved question that is why aspirin users have more polyps in total versus nonusers. Interestingly, most of the polyps developed from t1 to t2 in the distal but not in the proximal colon, which is in line with our molecular data showing that the protective effect of aspirin was found predominantly in the proximal colon. This relationship and general significance for broader population needs to be tested in follow-up studies.

**Fig. 4:**
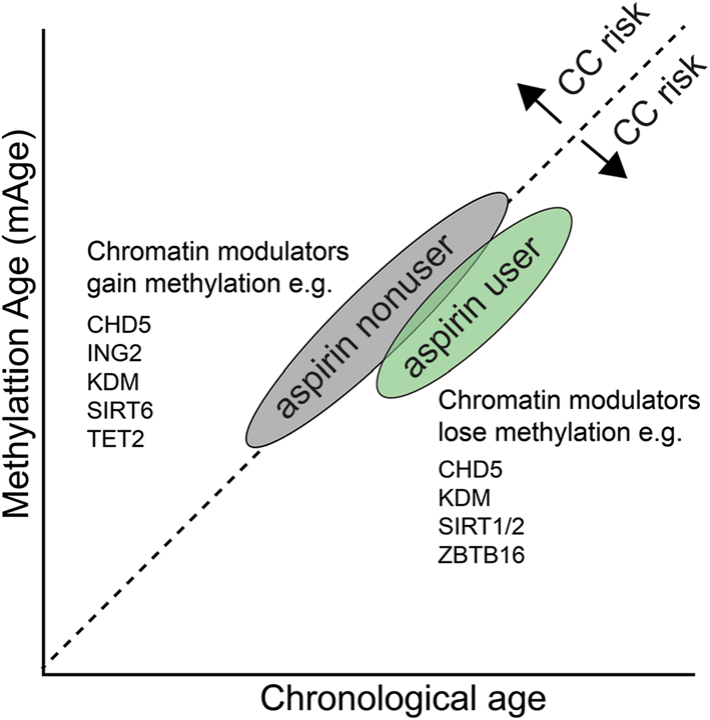
Schematic diagram illustrating the suppressed methylation age (mAge) in aspirin users. (↓) reduced colon cancer (CC) risk; (↑) increased colon cancer (CC) risk

Taken together, we show that methylation age in aspirin nonusers increases with increase in chronological age; however, in aspirin users, methylation age acceleration is suppressed (Fig. 4). Our data show that many CC-specific chromatin modulators gain methylation in nonusers over time (such as CHD5, PADI2, KDM8) but lose methylation in aspirin users, suggesting that the aspirin may reverse the hypermethylation of epigenetic modifiers and TSGs in colon. Investigating the effect of aspirin as an epigenetic modulator may help to better characterize the functional mechanisms underlying its anticancer activity.

## Conclusions

In conclusion, this is the first longitudinal study demonstrating a suppression of DNAm and epigenetic aging in healthy colon over time by aspirin use. While the study cohort is small and the observation period is short (10 years) in the context of a human lifespan, this pilot study has a strength of its longitudinal design. These findings provide a rationale for future longitudinal studies with large sample size to improve our understanding on epigenetic response to aspirin, optimize its potential benefit in the suppression of age-associated DNAm increase, CC risk, and hence increase its efficiency in CC prevention and therapy.

## Methods

### Study participants and data collection

We contacted 89 healthy females included in our previous study [17]. Colonoscopy could be performed in 31 females with 58 subjects being lost for various reasons (death, no response to invitation, discourage by family doctor for colonoscopy). Total 124 normal mucosa biopsies from cecum (proximal) and sigmoid (distal) colon were obtained at baseline (t1) and after 10 years (t2; range: 10–10.6 years) from 31 screening females (four biopsies per subject). Information on aspirin use was obtained using a self-administered questionnaire with assistance of a study nurse. Aspirin use was determined as never versus (vs.) long-term use (≥ twice per week for ≥ 2 years) at either the baseline (t1) or follow-up (t2). Of the 31 individuals, 17 were aspirin users at baseline and 20 at follow-up (three subjects switched from nonuser at t1 to user at t2). The term user was not restricted to the use of aspirin period immediately prior to the sampling at both time points. Height (cm) and weight (kg) were self-reported, and BMI was calculated (kg/m^2^). Polyp status was determined as no versus yes (any type) at baseline (t1) and at follow-up (t2). Polyp type was classified as tubular adenoma versus serrated lesions according to WHO classification [30].

### DNA isolation and bisulfite conversion

All endoscopies were performed by using high-definition colonoscopy including narrow-band imaging. Biopsies were taken from macroscopic normal mucosa without microscopically assessment of the collected specimens. Females with symptoms or history of inflammatory bowel disease were excluded from this study. Genomic DNA was extracted from freshly collected biopsies of normal mucosa stored in RNA*later* using Quick-DNA Miniprep kit (Zymo Research) including *RNAse A* treatment according to the manufacturer protocol. Five hundred ng of genomic DNA from each sample was bisulfite-treated using the EZ DNA Methylation kit (Zymo Research, Orange, CA), according to the manufacturer's protocol and stored at − 80 °C until further use.

### Genome-Wide DNA methylation profiling

Sodium bisulfite-treated DNA of 124 biopsies was subjected to measure global DNAm by Illumina infinium MethylationEPIC BeadChip [31]. Samples were randomly distributed across 21 chips (8 samples per chip) to avoid confounding batch effects. Illumina’s GenomeStudio software V2011.1 was used to extract the raw signal intensities of 865,859 probes. Downstream analysis was conducted using annotation developed previously mapping to hg38 or otherwise hg19 as indicated. Raw IDAT files were preprocessed using R/Bioconductor package minfi (version 1.32.0) [32, 33]. Samples were normalized using “preprocessFunnorm” including background subtraction and dye-bias normalization. Methylation levels were computed as Beta (*β*) values (Meth/[Meth + Unmeth + 100]) and as logit-transformed *M* value (log2 [Meth/Unmeth]). To avoid extreme *M* values, we use beta threshold of 1e-07 to make sure beta was neither 0 nor 1, before taken the logit. Probes where at least one sample had a detection *P* value > 0.01 and the probes recommended previously [34] using hg38 genome as general-purpose masking probes were filtered out. After filtering, 753,630 probes were kept for further analysis. *M* value were used to identify differentially methylated CpGs (dmCpGs) using bioconductor *limma* [35] package.

### Epigenetic aging-clocks

To derive an estimate of DNA methylation Age (mAge), we used three different algorithms: the blood based Hannum clock [22], multi-tissue based Horvath clock [21] and blood based PhenoAge [23] that differentiate morbidity and mortality risk among same-age individuals were used to determine epigenetic aging. Of the requisite 71 CpGs in Hannum-CpGs, 6 are missing from the EPIC and 4 were filtered out at QC. Of the requisite 353 CpGs in Horvath-CpGs, 17 are missing from EPIC and the 10 were filtered out during data quality control (QC). Of the requisite 513 CpGs in PhenoAge clock, 18 were filtered out during data QC. The methylation levels on the remaining 61 of Hannum-CpGs, 326 Horvath-CpGs and 495 PhenoAge CpGs were utilized to compare median methylation differences in Fig. 3a. The mAge and age acceleration residuals (AAR) for three algorithms were calculated using the software (https://dnamage.genetics.ucla.edu/new). The raw beta values without masking probes based on a detection *P* value were submitted. This resulted in submission of 64 of Hannum-CpGs, 336 Horvath-CpGs and 513 PhenoAge CpGs. The age calculator automatically imputes missing data across platform based on k-nearest neighbor imputation.

### Pathway analysis and annotation

Pathway analysis was done using Kyoto Encyclopedia of Genes and Genomes (KEGG). Gene annotation was obtained from IlluminaHumanMethylationEPICanno.ilm10b4.hg19, and number of CpGs per gene was controlled. For enrichment, all genes present on BeadChip were used as background and pathways < 0.05 adjusted *P* value were considered. For functional annotation, TSGene [25], ONGene [24] and EpiFactors [36] database were used. Detailed annotation of probes on gene regulatory elements (promoters, enhancers and weak transcripts) were done using ENCODE [37] as described previously [34]. The CpG Island (CGI) were defined as in UCSC.

### Statistical analyses

Normalized methylation levels were analyzed using multivariable linear regression model between t2 versus t1 adjusted for body mass index (BMI), polyps, age and batch-effect. The paired nature of samples was taken into account by adding subjects in the model matrix. First, model matrix was designed to compare aspirin users to nonusers for each time point and colon location, and then, contrasts were made to address the effect of aspirin in user and nonuser from t1 to t2. Differentially methylated CpGs (dmCpGs) were identified using empirical Bayes moderated *t* statistics and associated Benjamini–Hochberg (BH) false discovery rate (FDR) adjusted *P* values < 0.05 were used as cutoff. Shapiro–Wilk test was used for normality testing for Fig. 1d and 3a. For methylation index (MI) analysis in Fig. 1d, paired Wilcoxon signed rank test was used. Wilcoxon rank sum tests were used to determine significance of median methylation differences between groups at each epigenetic clock in Fig. 3a. For enrichment analysis, *P* values were calculated using Fisher’s test. All analyses were performed using *R* (version 4.0.2) and R-Studio (version 1.1.463).

## Supplementary information

**Additional file 1.** **Figure S1. a)** Overlap of all significant differentially methylated CpGs found in aspirin users and nonusers. The non-overlapping CpGs were then defined as aspirin user specific differentially methylated CpGs (U-dmCpGs) or nonuser specific differentially methylated CpGs (Nu-dmCpGs). **b)** Overlap of genes affected by Nu-dmCpGs and U-dmCpGs with known differential expressed colon cancer specific tumor suppressor genes (TSGs) and oncogenes. Shown are separate overlaps for proximal and distal colon.

## Data Availability

The DNA methylation data generated in this study are available through NCBI Gene Expression Omnibus (GEO) https://www.ncbi.nlm.nih.gov repository under the Accession Number GSE142257.

## Abbreviations

CC: Colon cancer
DNAm: DNA methylation
mAge: Methylation age
U: Aspirin users
Nu: Aspirin nonusers
U-dmCpGs: Differentially methylated CpGs in aspirin users
Nu-dmCpGs: Differentially methylated CpGs in nonusers
hyper: Hypermethylation
hypo: Hypomethylation
MI: Methylation index
CGI: CpG-island
TSG: Tumors suppressor genes
t1: Time point 1
t2: Time point 2
