# Supplementary material for: Longitudinal analysis of healthy colon establishes aspirin as a suppressor of cancer-related epigenetic aging
Source: Clin Epigenetics. 2020 Nov 3;12:164. doi: 10.1186/s13148-020-00956-9 (PMC7607658; doi:10.1186/s13148-020-00956-9)
Supplement: Supplementary file 1 — Additional file 1. Figure S1. a) Overlap of all significant differentially methylated CpGs found in aspirin users and nonusers. The non-overlapping CpGs were then defined as aspirin user specific differentially methylated CpGs (U-dmCpGs) or nonuser specific differentially methylated CpGs (Nu-dmCpGs). b) Overlap of genes affected by Nu-dmCpGs and U-dmCpGs with known differential expressed colon cancer specific tumor suppressor genes (TSGs) and oncogenes. Shown are separate overlaps for proximal and distal colon. [file 13148_2020_956_MOESM1_ESM.docx]

**Additional file 1**

**Longitudinal analysis of healthy colon establishes aspirin as a suppressor of cancer-related epigenetic aging**

**Authors and affiliations**

Faiza Noreen, PhD^1,2,*^, Anna Chaber-Ciopinska, PhD^3^, Jaroslaw Regula, MD, PhD^3^, Primo Schär, PhD^1^ , Kaspar Truninger, MD^1,4*^

^1^Department of Biomedicine, University of Basel, Basel, 4058, Switzerland; ^2^Swiss Institute of Bioinformatics, Basel, 4053, Switzerland; ^3^Department of Gastroenterology, Medical Center for Postgraduate Education and Maria Sklodowska-Curie Memorial Cancer Center, Warsaw, Poland; ^4^Gastroenterologie Oberaargau, Langenthal, 4900, Switzerland

**Additional file 1: Figure S1**


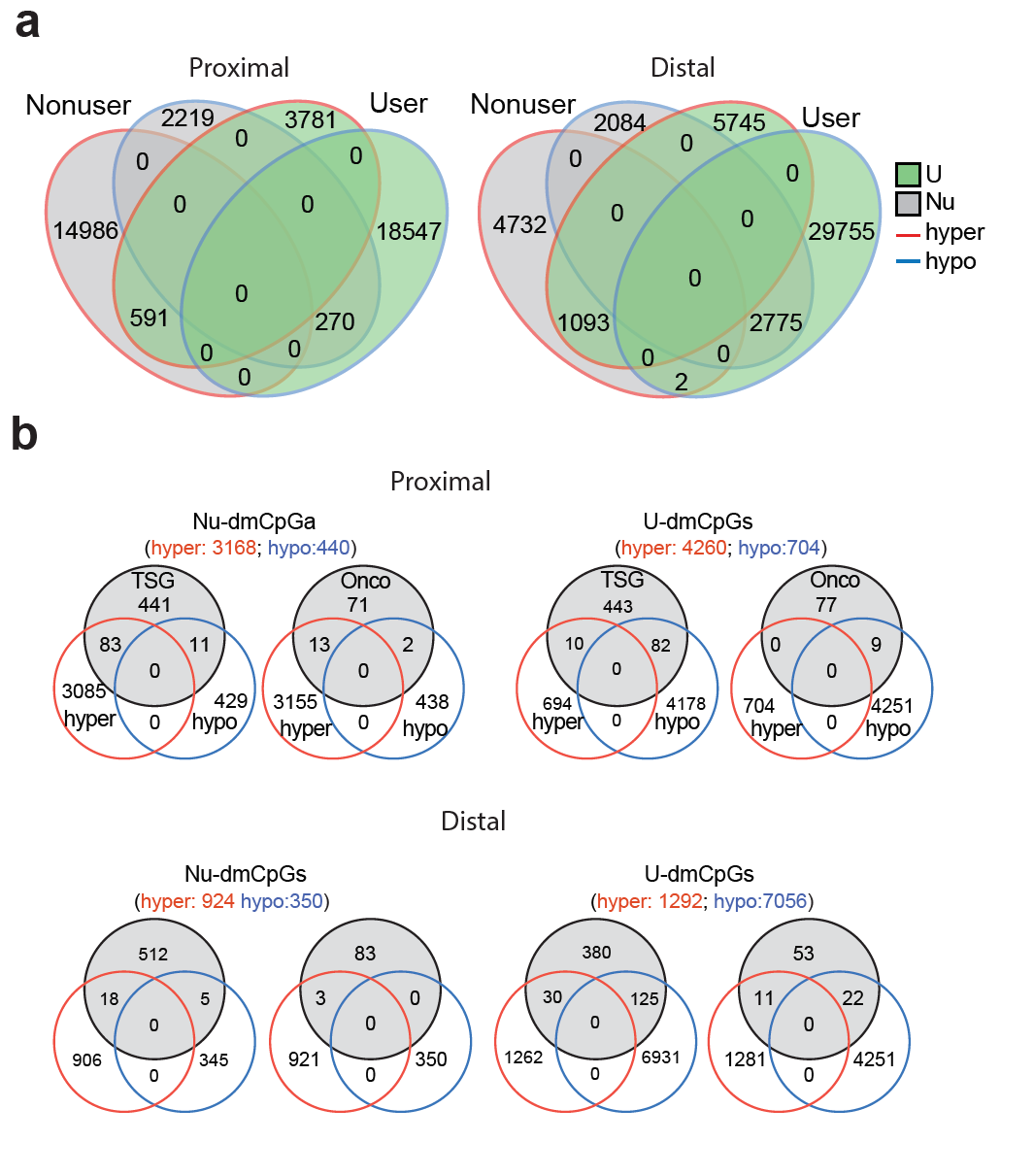


**Additional file 1: Figure S1. a**) Overlap of all significant differentially methylated CpGs found in aspirin users and nonusers. The non-overlapping CpGs were then defined as aspirin user specific differentially methylated CpGs (U-dmCpGs) or nonuser specific differentially methylated CpGs (Nu-dmCpGs). **b**) Overlap of genes affected by Nu-dmCpGs and U-dmCpGs with known differential expressed colon cancer specific tumor suppressor genes (TSGs) and oncogenes. Shown are separate overlaps for proximal and distal colon.
